# Synchronous double primary malignancies of the pancreatic body and extrahepatic bile duct treated with pancreatoduodenectomy and splenic artery resection following neoadjuvant chemotherapy with gemcitabine plus nab-paclitaxel: a case report

**DOI:** 10.1186/s40792-022-01383-z

**Published:** 2022-02-16

**Authors:** Takahiro Murokawa, Takehiro Okabayashi, Kenta Sui, Motoyasu Tabuchi, Jun Iwata

**Affiliations:** 1 Department of Gastroenterological Surgery at Kochi Health Sciences Center, 2125-1 Ike, Kochi-City, Kochi 781-8555 Japan; 2 Department of Diagnostic Pathology at Kochi Health Sciences Center, 2125-1 Ike, Kochi-City, Kochi 781-8555 Japan

**Keywords:** Double primary malignancy, Pancreatic cancer, Extrahepatic bile duct cancer, Neo-adjuvant chemotherapy

## Abstract

**Background:**

Primary pancreatic cancer with synchronous primary tumors in other organs is a rare condition, and its treatment largely depends on the progression of pancreatic cancer. Here, we describe a rare case of double primary malignancies involving borderline resectable pancreatic body and extrahepatic bile duct cancers that were successfully resected after neoadjuvant chemotherapy (NAC), subsequently avoiding total pancreatectomy.

**Case presentation:**

A 61-year-old Japanese male was referred to our hospital by his general practitioner after presenting with elevated liver enzymes during a routine check-up for type 2 diabetes mellitus. He was diagnosed with synchronous borderline resectable pancreatic cancer in the body of the pancreas and lower extrahepatic bile duct cancer with obstructive jaundice. Abdominal computed tomography (CT) confirmed a hypovascular mass in the pancreatic body with partial encasement of the common hepatic artery, left gastric artery, celiac artery, and splenic artery and invasion of the splenic vein. Endoscopic retrograde cholangiopancreatography and bile duct biopsy confirmed lower bile duct cancer. Following multidisciplinary discussion, endoscopic retrograde biliary drainage was performed, and neoadjuvant chemotherapy comprising gemcitabine plus nanoparticle albumin-bound paclitaxel (GEM + nab-PTX) was administered. After a total of seven cycles of chemotherapy, follow-up CT showed that the size of the pancreatic lesion reduced, following which the patient underwent pancreatoduodenectomy with splenic artery resection. The postoperative course was uneventful without any surgical complications or intensive hypoglycemic treatment. The pathological diagnosis was pancreatic ductal adenocarcinoma (ypT3N1aM0 ypStage IIB/UICC 8th) with synchronous extrahepatic cholangiocarcinoma (ypT2N1M0 ypStage IIB/UICC 8th). R0 pancreatic resection was performed with an Evans grade III response to neoadjuvant chemotherapy. The patient was followed up and had no tumor recurrence at 22 months after surgery with adjuvant S-1 chemotherapy, however, died after 32 months after surgery due to multiple liver metastasis and para-aortic lymph node metastasis despite salvage GEM + nab-PTX chemotherapy.

**Conclusion:**

In our case, neoadjuvant chemotherapy for borderline resectable pancreatic cancer and function-preserving pancreatoduodenectomy (R0 resection) for double primary malignancies achieved balanced patient survival and postoperative quality of life.

## Background

Synchronous double primary malignancies are more frequently detected owing to progress in imaging modalities and an increasing life expectancy [1]. Pancreatic cancer with synchronous or metachronous primary malignancies in other organs is relatively rare, with a reported frequency of 0.75–20.0% [2, 3]. Double primary malignancies of the pancreas and common bile duct are rarely encountered [4–6]. The prognosis of patients with pancreatic cancer and co-occurrence of double primary malignancies largely depends on the prognosis of the pancreatic malignancy [7]. Therefore, therapeutic interventions for pancreatic cancer should be considered more important than those for extrapancreatic cancers [5]. Radical excision remains the only curative treatment for patients with pancreatic cancer. Synchronous double primary malignancies of the pancreatic body and extrahepatic bile duct usually require total pancreatectomy to achieve curative resection. While total pancreatectomy can be safely performed, decreased postoperative quality of life (QOL) because of pancreatic endocrine insufficiency is a major drawback [8].

Approximately 30–40% of patients with pancreatic cancer are diagnosed with “borderline resectable” or “locally advanced pancreatic cancer”. This indicates a poor prognosis owing to a low R0 resection rate if upfront surgical resection is performed. However, recent potent oncological regimens, such as FOLFIRINOX or gemcitabine (GEM) plus nanoparticle albumin-bound paclitaxel (nab-PTX), considerably control local cancer progression, leading to conversion surgery with curative resection [9, 10].

In this report, we describe a rare case of synchronous double primary malignancies involving borderline resectable pancreatic body cancer and extrahepatic bile duct cancer that were curatively resected via pancreatoduodenectomy with splenic artery resection (PD-SAR) following neoadjuvant chemotherapy (NAC).

## Case presentation

**Fig. 1:**
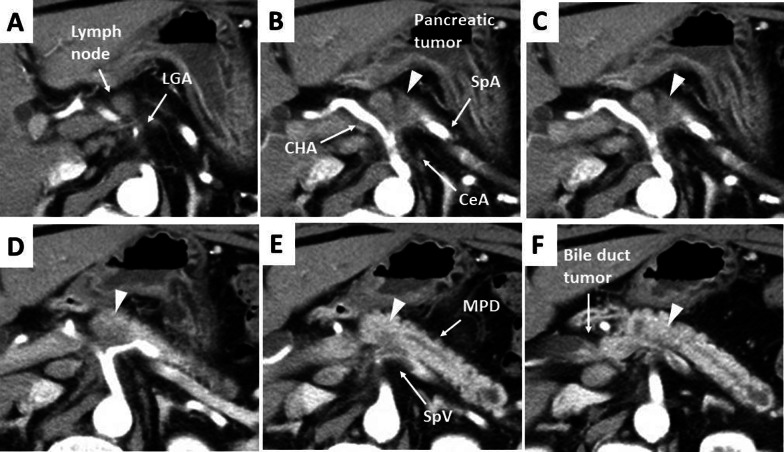
Abdominal contrast computed tomography (CT) findings at initial diagnosis. **A** The left gastric artery (LGA) was surrounded by soft tissue. Swollen regional Lymph node was adjacent to the pancreatic tumor. **B–D** 40-mm-sized hypovascular mass in the pancreatic body that was radiologically consistent with pancreatic ductal adenocarcinoma (arrowhead) showing wide abutment the common hepatic artery and the splenic artery (SpA). **C** Hazy attenuation connected to the tumor was seen on the Celiac artery. **D** Contour irregularity was seen on SpA and focal narrowing was seen on the splenic vein (SpV). **E** The main pancreatic duct (MPD) was slightly dilated in the distal side. **F** the lower bile duct tumor was enhanced

**Fig. 2:**
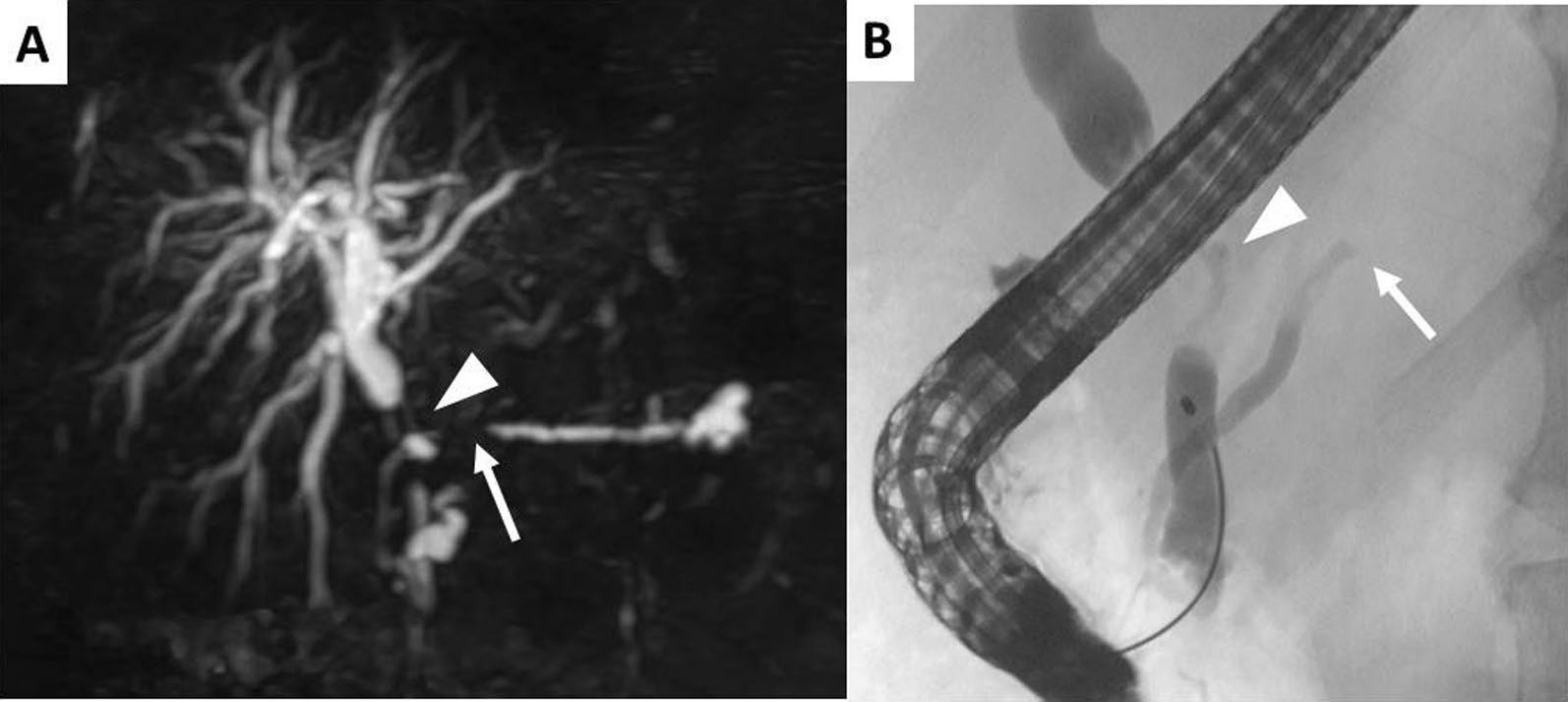
**A** Magnetic resonance cholangiopancreatography findings (MRCP). **B** Endoscopic retrograde cholangiopancreatography (ERCP) findings. The MRCP and ERCP revealed the disruption of the main pancreatic duct in the body of the pancreas (arrow) and unilateral narrowing of the lower common bile duct (arrowhead). The image of MRCP showed the slight dilatation of the main pancreatic duct (3.4 mm) in the distal side

**Fig. 3:**
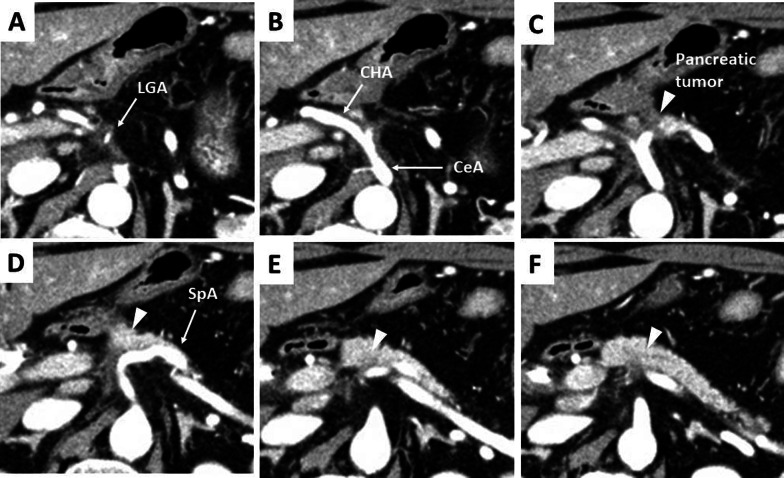
A computed tomography (CT) findings after five cycles of chemotherapy. **A**, **B** The soft tissue mass surrounding the left gastric artery (LGA) and CeA slightly remained. **C–F** The pancreatic tumor (arrowhead) and lymph node shrunk showing improvement in the deformity of the splenic artery (SpA) and involvement of the celiac artery (CeA) and common hepatic artery (CHA). **E**, **F** The dilatation of the main pancreatic duct was disappeared

**Fig. 4:**
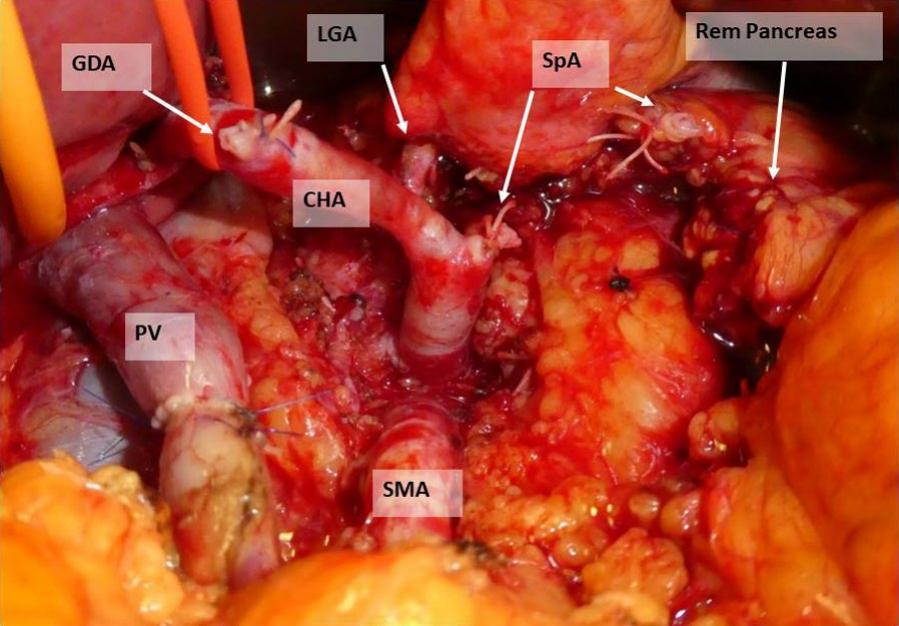
Intraoperative findings after Pancreatoduodenectomy with splenic artery resection (PD-SAR). *GDA*: gastroduodenal artery, *PV*: portal vein, *CHA*: common hepatic artery, *LGA*: left gastric artery, *SMA*: superior mesenteric artery, *SpA*: splenic artery, *Rem pancreas*: remnant pancreas

**Fig. 5:**
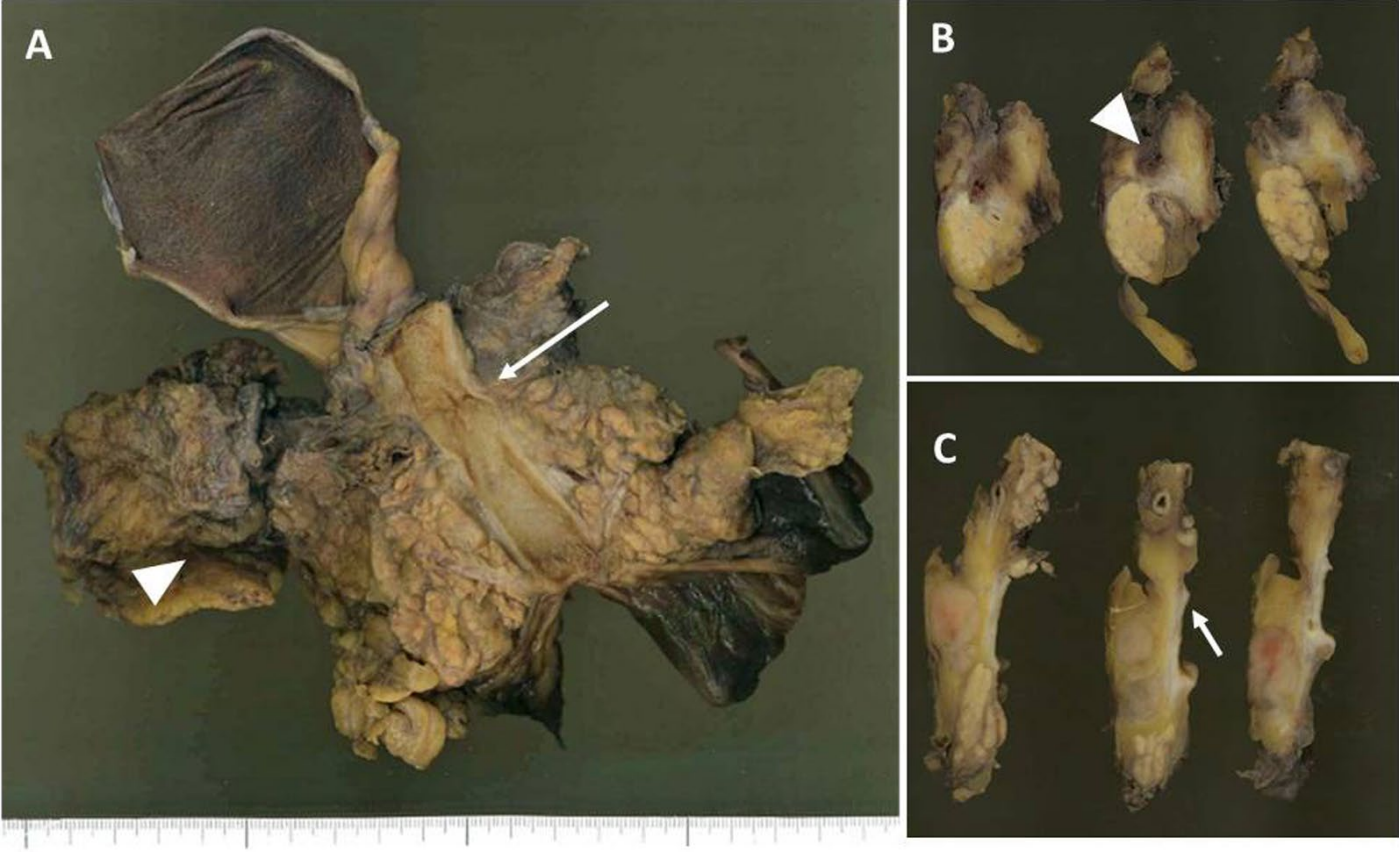
Pancreatoduodenectomy with splenic artery resection (PD-SAR) was performed. **A** The resected specimen revealed two tumors in the body of the pancreas (arrowhead) and lower common bile duct (arrow). **B** The representative cut surfaces of the resected specimen of the pancreas showed a 38-mm-sized mass surrounded by fibrosis in the pancreatic body. **C** The representative cut surfaces of the resected specimen of the common bile duct showed an infiltrating-type flat tumor in the lower bile duct with several para-pancreatic lymph node metastases

**Fig. 6:**
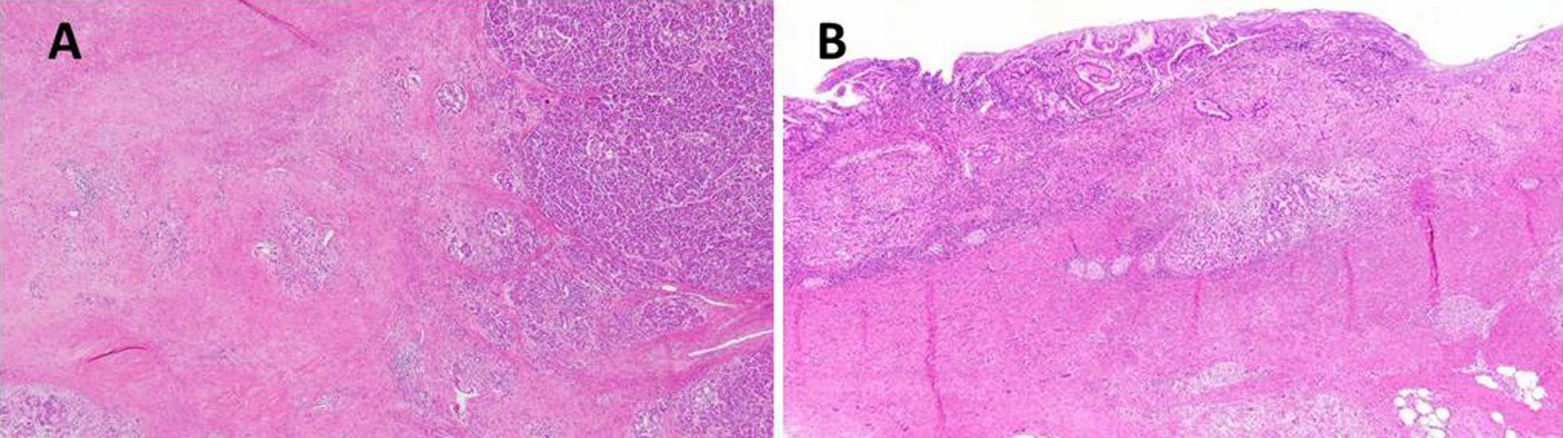
Histological examination. **A** Moderately differentiated tubular adenocarcinoma with marked degeneration was present in the pancreatic body. Only 10% of the viable tumors were residual and defined as grade III according to the Evans classification. **B** Non-solid type, poorly differentiated adenocarcinoma in the extrahepatic duct with Evans grade IIb tumor destruction

A 61-year-old Japanese male was referred to our hospital due to abnormal liver function tests without abdominal pain during a routine check-up for type 2 diabetes mellitus. He had history of alcoholic liver disease and hyperuricemia. He did not have a personal or family history of cancer. Laboratory data revealed marked elevations of liver and biliary tract enzymes with slight jaundice. Level of cancer antigen 19-9 (Ca19-9) was elevated while that of CEA was within normal range (Table 1). Abdominal contrast computed tomography (CT) revealed a 40-mm-sized hypovascular mass in the pancreatic body, which was radiologically consistent with pancreatic ductal adenocarcinoma (Fig. 1A–F). The pancreatic tumor and adjacent lymph node together contacted bifurcation of the celiac artery (CeA), common hepatic artery (CHA), splenic artery (SpA) and left gastric artery (LGA) (Fig. 1A, B). Increased hazy attenuation on CeA was present ≦180 (Fig. 1B, C). There was contour irregularity in SpA showing tumor invasion (Fig. 1D). The main pancreatic duct was slightly dilated (3.4 mm) (Fig. 1E). Focal narrowing was seen in Splenic vein (Fig. 1F). He underwent magnetic resonance cholangio pancreatography and endoscopic retrograde cholangiopancreatography (ERCP) and confirmed disruption of the main pancreatic duct in the body of the pancreas with slight main pancreatic duct dilatation and unilateral narrowing over 3 cm long of the lower common bile duct (Fig. 2A, B). Endoscopic transpapillary bile duct biopsy resulted poorly differentiated adenocarcinoma of the extrahepatic bile duct. Endoscopic retrograde biliary drainage was also performed. He was radiographically staged with T4N1M0 borderline resectable pancreatic cancer (UICC 8th) and T1N1M0 lower bile duct cancer (UICC 8th) received chemotherapy targeting pancreatic cancer (GEM, 1600 mg/m^2^ body surface area plus nab-PTX, 200 mg/m^2^ body surface area on days 1 and 8 of a 21-day cycle). At diagnosis the CA19-9 level was remarkably high at 1266 U/mL; this level decreased to 186 U/ml after ERBD. After two cycles of induction chemotherapy with GEM plus nab-PTX, level of CA19-9 resolved to the normal range at 34.8 U/mL. The pancreatic lesion showed shrinkage after five cycles of chemotherapy, improving the deformity of the SpA and SpV, the involvement of CeA and the dilatation of MPD (Fig. 3A–F). The level of CA19-9 dropped further to 10.4 U/mL after total of seven cycles of chemotherapy. Seven months after diagnosis, we performed pylorus-preserving PD-SAR, subsequently avoiding total pancreatectomy (Fig. 4). The proximal part of SpA was resected with the pancreatic tumor: SpA was resected at its root and the level where the posterior gastric artery was preserved. Since LGA could be isolated and preserved, total gastrectomy was not required. SpV was partially resected with the tumor. Portal veins had to be resected and reconstructed for its kinking after SpV resection. The surgical duration was 430 min with a blood loss of 1800 mL. Macroscopic examination of the surgical specimen revealed a 38-mm-sized firm mass in the body of the pancreas (Fig. 5A, B) and a 43-mm-sized flat infiltrative tumor in the lower extrahepatic bile duct (Fig. 5A, C). Microscopic examination of the pancreatic lesion showed a moderately differentiated pancreatic ductal adenocarcinoma with remarkable fibrosis in the pancreatic body. The tumor degeneration grade was Evans grade III (Fig. 6A) [11]. Histologically, there was no invasion into the SpA; however, venous and perineural invasions were noted. Two para-pancreatic lymph nodes had metastasized. The final diagnosis of pancreatic tumor was ypT2ypN1M0, ypStage IIB pancreatic cancer (UICC 8th). There were no residual cancer cells in the dissected margin and/or pancreatic resection margin (R0). Synchronous poorly differentiated adenocarcinoma in the distal bile duct was also pathologically diagnosed as ypT2(SS)ypN1M0, ypStage IIB (UICC 8th). The therapeutic response was Evans grade IIa (Fig. 6B). These two tumors showed no histopathological continuity, and according to the Warren and Gates criteria [12], the tumors were classified as double primary malignancies. The patient’s postoperative course was uneventful, and he was discharged on postoperative day 16. Although intensive insulin therapy was not required in the perioperative period, insulin or oral hypoglycemic drugs were required to maintain blood glucose levels 4 months after discharge. The patient received adjuvant chemotherapy with S-1 (TS-1; Taiho Pharmaceutical Co., Ltd., Tokyo, Japan) one month after surgery. No signs of recurrence have been observed for 22 months after surgery with continuing S-1. However, he developed multiple liver metastasis and para-aortic LN metastasis. GEM + nab-PTX was reintroduced as salvage chemotherapy. He had seven cycles of that chemotherapy, but he died 32 months after surgery.

**Table 1:** Laboratory data

Laboratory test	Value	Unit
White blood cells	5900	/μL
Red blood cells	473 × 10^4^	/mm^3^
Hemoglobin	14.5	g/dL
Hematocrit	41.1	%
Platelets	21.9 × 10^4^	mm^3^
Blood urea nitrogen	8.5	mg/dL
Creatinine	0.6	g/dL
Total protein	7.5	g/dL
Albumin	4.7	g/dL
Total bilirubin	1.6	mg/dL
Aspartate aminotransferase	474	U/L
Alanine aminotransferase	966	U/L
Lactate dehydrogenase	410	U/L
Alkali phosphatase	1430	U/L
Gamma-glutamyl transpeptidase	3290	U/L
Amylase	134	U/L
C-reactive protein	0.5	mg/dL
Hemoglobin A1c	6.5	%
CA19-9	1266	U/mL
CEA	1.2	ng/mL

*CA19-9* cancer antigen 19-9, *CEA* carcinoembryonic antigen

## Discussion

Here, we report a rare case of double primary malignancies of the pancreatic body and common bile duct. Although the pancreatic lesion was initially considered borderline resectable cancer, R0 resection was achieved owing to NAC. We avoided total pancreatectomy by performing PD-SAR to balance curability with the patient’s postoperative QOL.

Pancreatic cancer is a devastating malignancy with poor prognosis. The 5-year overall survival rate is as low as 8% [13]. Radical resection is considered the only therapeutic option to cure pancreatic cancer, although many cases of severe loco-regional invasion and/or distant metastasis at initial diagnosis have been noted. However, recent studies have demonstrated that the modern potent chemotherapy protocol, which includes FOLFIRINOX (fluorouracil, leucovorin, irinotecan, and oxaliplatin) or GEM plus nab-PTX, improved responses for not only metastatic tumors, but also more anatomically advanced cases [14, 15].

According to recent literature, co-occurring multiple primary malignancies in patients with pancreatic cancer were occasionally detected with an incidence between 5.3% and 13.8% [4–6, 16–18]. Intra-papillary mucinous neoplasms are frequently associated with a higher risk of carcinogenesis in other organs. In addition, genetic disorders, including hereditary pancreatitis, Lynch syndrome, and Peutz–Jeghers syndrome, are associated with 10% of pancreatic cancers and may increase the risk of secondary malignancies in other organs. Organs typically associated with primary malignancies include the stomach (22–40%), colorectum (20–40%), lungs (14.9–31.2%), thyroid (10.7–20%), and prostate (12.5–17.2%) [4–6, 16–18]. Double primary malignancies of the pancreas and extrahepatic bile duct are extremely rare. When reviewing English literature, very few cases reported about the resection of multiple primary malignancies of the pancreas and bile duct [19–21], in which extrahepatic bile duct cancer was incidentally diagnosed after surgery. In a series of 113 curatively resected cases of pancreatic cancer with double primary malignancies, only two cases (1.7%) of distal bile duct cancer were reported (6). The overall survival of patients with pancreatic cancer and double primary malignancies largely depends on the progression of the pancreatic lesion. Few studies have analyzed the postoperative course of multiple double primary malignancies involving a pancreatic cancer. Yamamura et al. reported that there was no significant difference between the solitary pancreatic cancer group and multiple primary malignancies involving pancreatic cancer in terms of overall survival [5]. As a result, the principle of treatment for multiple malignancies involving pancreatic cancer has been complete surgical resection of the pancreas. If surgery is not an option, a chemotherapy regimen for pancreatic cancer is selected. Clinical practice for extrapancreatic cancers is determined based on the progression of the pancreatic cancer and the patients’ general condition [17]. In our case, surgery was the only treatment that could cure both pancreatic and extrahepatic bile duct cancers. However, the pancreatic cancer presented with a wide abutment of the CHA, SpA, and CeA and was thus initially diagnosed as borderline resectable pancreatic cancer. Therefore, we selected NAC to primarily target the pancreatic cancer.

Surgical procedures for pancreatic cancer include pancreatoduodenectomy, distal pancreatectomy, and total pancreatectomy. The type of procedure performed is dependent on the tumor location and likelihood of achieving R0 resection. Generally, surgeons are reluctant to perform total pancreatectomy as it decreases a patient’s QOL [8]. We performed PD-SAR to preserve as much pancreatic endocrine and exocrine function as possible [22, 23]. Pancreatoduodenectomy with SpA resection was firstly reported by Desaki et al. and was inspired by Sutherland et al.’s and Warshaw’s technique, which involves spleen preservation during distal pancreatectomy. The splenic artery and vein are resected together with the pancreas while carefully preserving the vascular collaterals in the splenic hilum. During our surgery, after cutting the pancreatic parenchyma when removing the tumor, blood flow was observed from the remnant pancreatic stump. This implied that blood flow was supplied by the LGA via the left gastroepiploic artery, posterior gastric artery, and posterior epiploic artery. Sufficient blood flow to the remnant pancreas may confirm the absence of anastomotic leakage and low insulin requirements during the perioperative period.

The positive response of the bile duct tumor and the pancreatic lesion to the NAC was another miscellaneous finding of this case. Interestingly, tumor regression was evident in the bile duct in response to the GEM-based regimen, despite anticipating a poor outcome. In the BILCAP study, adjuvant chemotherapy for biliary tract cancers resulted in a longer median overall survival time in the capecitabine group than in the observation group in the per-protocol analysis (but not in the intention-to-treat analysis) [24]. In contrast, to our knowledge, there are not much data regarding the use of preoperative chemotherapy for downstaging biliary tract cancers. The use of NAC in conjunction with postoperative chemotherapy for biliary tract cancers also requires further investigation.

In summary, we described an extremely rare case of borderline resectable pancreatic cancer in the pancreatic body with synchronous extrahepatic bile duct cancer. Pancreatoduodenectomy with SpA resection following GEM plus nab-PTX chemotherapy may assist in balancing operative radicality with postoperative QOL for selected advanced hepatobiliary double malignancies. Where possible, when treating devastating pancreatic cancer and performing R0 resections, preserving remnant pancreatic function may be more patient friendly.

## Data Availability

All data generated or analyzed during this study are included in the published article.
